# The beneficial effects of Tai Chi exercise on endothelial function and arterial stiffness in elderly women with rheumatoid arthritis

**DOI:** 10.1186/s13075-015-0893-x

**Published:** 2015-12-24

**Authors:** Jeong-Hun Shin, Yonggu Lee, Soon Gil Kim, Bo Youl Choi, Hye-Soon Lee, So-Young Bang

**Affiliations:** Division of cardiology, Department of Internal Medicine, College of Medicine, Hayang University, Kyoungchun-ro 153, Guri, Kyeonggi-do 11923 Republic of Korea; Division of cardiology, Department of Internal Medicine, Sung-Ae Hospital, 22 Yoidaebang-ro 53 Road, Yongdeungpo-gu, Seoul, 07354 Republic of Korea; Department of Preventive Medicine, College of Medicine, Hanyang University, 222-1, Wangsimni-ro, Seongdong-gu, Seoul, 04763 Republic of Korea; Division of Rheumatology, Department of Internal Medicine, College of Medicine, Hanyang University, Kyoungchun-ro 153, Guri, Kyeonggi-do 11923 Republic of Korea

**Keywords:** Rheumatoid arthritis, Tai Chi, Cardiovascular risk

## Abstract

**Background:**

Rheumatoid arthritis (RA) has been known to be associated with increased risk of cardiovascular disease (CVD). The aim of this study was to investigate the effects of Tai Chi exercise on CVD risk in elderly women with RA.

**Method:**

In total, 56 female patients with RA were assigned to either a Tai Chi exercise group (29 patients) receiving a 3-month exercise intervention once a week or a control group (27 patients) receiving general information about the benefits of exercise. All participants were assessed at baseline and at 3 months for RA disease activity (Disease Activity Score 28 and Routine Assessment of Patient Index Data 3), functional disability (Health Assessment Questionnaire), CVD risk factors (blood pressure, lipids profile, body composition, and smoking), and three atherosclerotic measurements: carotid intima-media thickness, flow-mediated dilatation (FMD), and brachial-ankle pulse wave velocity (baPWV).

**Results:**

FMD, representative of endothelial function, significantly increased in the Tai Chi exercise group (initial 5.85 ± 2.05 versus 3 months 7.75 ± 2.53 %) compared with the control group (initial 6.31 ± 2.12 versus 3 months 5.78 ± 2.13 %) (*P* = 1.76 × 10^−3^). Moreover, baPWV, representative of arterial stiffness, significantly decreased in the Tai Chi exercise group (initial 1693.7 ± 348.3 versus 3 months 1600.1 ± 291.0 cm/s) compared with the control group (initial 1740.3 ± 185.3 versus 3 months 1792.8 ± 326.1 cm/s) (*P* = 1.57 × 10^−2^). In addition, total cholesterol decreased significantly in the Tai Chi exercise group compared with the control group (−7.8 ± 15.5 versus 2.9 ± 12.2 mg/dl, *P* = 2.72 × 10^−2^); other changes in RA-related characteristics were not significantly different between the two groups. Tai Chi exercise remained significantly associated with improved endothelial function (FMD; *P* = 4.32 × 10^−3^) and arterial stiffness (baPWV; *P* = 2.22 × 10^−2^) after adjustment for improvement in total cholesterol level.

**Conclusion:**

Tai Chi exercise improved endothelial dysfunction and arterial stiffness in elderly women with RA, suggesting that it can be a useful behavioral strategy for CVD prevention in patients with RA.

**Electronic supplementary material:**

The online version of this article (doi:10.1186/s13075-015-0893-x) contains supplementary material, which is available to authorized users.

## Background

Rheumatoid arthritis (RA) (MIM 180300) is a chronic systemic inflammatory disease characterized by articular and extra-articular involvement. It associates with high cardiovascular disease (CVD) morbidity, which is not fully explained by the presence of traditional CVD risk factors [1–4]. Recently, there has been growing interest in the prevention of CVD in patients with RA. Because many patients with RA have below-average levels of physical activity with a sedentary lifestyle, appropriate exercise training should be included as an important treatment modality of RA. Moreover, given that the main cause of reduced life expectancy in RA is CVD-related, the probable cardioprotective benefit of regular exercise to patients with RA cannot be ignored. To date, however, most studies of the beneficial effects of exercise training in RA have focused on improvements in functional ability and other RA-related disease activity. Few studies have explored the cardiovascular benefits of exercise for RA patients, who already have higher cardiovascular risk as well as lower baseline levels of activity [5].

Several studies have suggested that the development of atherosclerosis, the underlying process of CVD, is increased in RA [6–9]. Atherosclerosis is a dynamic inflammatory process that begins with the activation of the vascular endothelium, immigration of leukocytes, and lipid oxidation and culminates with plaque destabilization and thrombosis. Striking similarities have been noted between the inflammatory pathways in atherosclerosis and those in RA [10, 11]. In patients with RA, the result of increased systemic inflammation leads to a pro-atherogenic profile, namely, endothelial dysfunction and increased arterial stiffness [6]. Endothelial dysfunction is a pivotal early step in atherosclerosis and is measured non-invasively by brachial artery flow-mediated dilatation (FMD). Arterial stiffness is also an important indicator of vascular disease and is measured non-invasively by brachial ankle pulse wave velocity (baPWV). Previous studies demonstrated that endothelial dysfunction and arterial stiffness were predictors of adverse cardiovascular events [12, 13], which were improved by medical therapy [14] or exercise [15–17]. Moreover, improvement in endothelial function after aerobic exercise has been recently shown in patients with RA [18].

Tai Chi, a set of Chinese systematic callisthenic exercises, combines deep breathing and relaxation with slow, relaxed, continuous movements and is officially supported by the Arthritis Foundation of Australia as a complementary therapy for RA. As a form of physical exercise, Tai Chi enhances cardiovascular fitness, muscular strength, balance, and physical function. It also appears to be associated with reduced stress, anxiety, and depression as well as improved quality of life [19]. In patients with RA, a randomized controlled trial pilot study showed that Tai Chi reduced RA symptoms, disease activity, and improved quality of life [20].

However, the effect of Tai Chi exercise on endothelial function and arterial stiffness in RA has not been studied. The aim of the present study was to investigate the effects of Tai Chi exercise on CVD risk, including arterial stiffness and endothelial function, in elderly women with RA.

## Methods

### Participants

Female patients with RA were consecutively recruited from the rheumatology department of Hanyang University Guri Hospital, and all satisfied the American College of Rheumatology 1987 revised classification criteria for RA [21]. Inclusion criteria were more than 50 years old, sedentary lifestyle (no participation in structured exercise for the preceding 6 months), and stable disease (no changes in disease-modifying anti-rheumatic drugs (DMARDs) or steroid in the last 3 months). Patients with an inability to bear weight on the lower extremities, recent or ongoing disease flare, unstable heart conditions (including atrial fibrillation and heart failure), or serious comorbidities such as terminal malignancy were excluded. During the study period, medical treatments for RA such as DMARDs or steroid administration at baseline were maintained without any change.

**Fig. 1:**
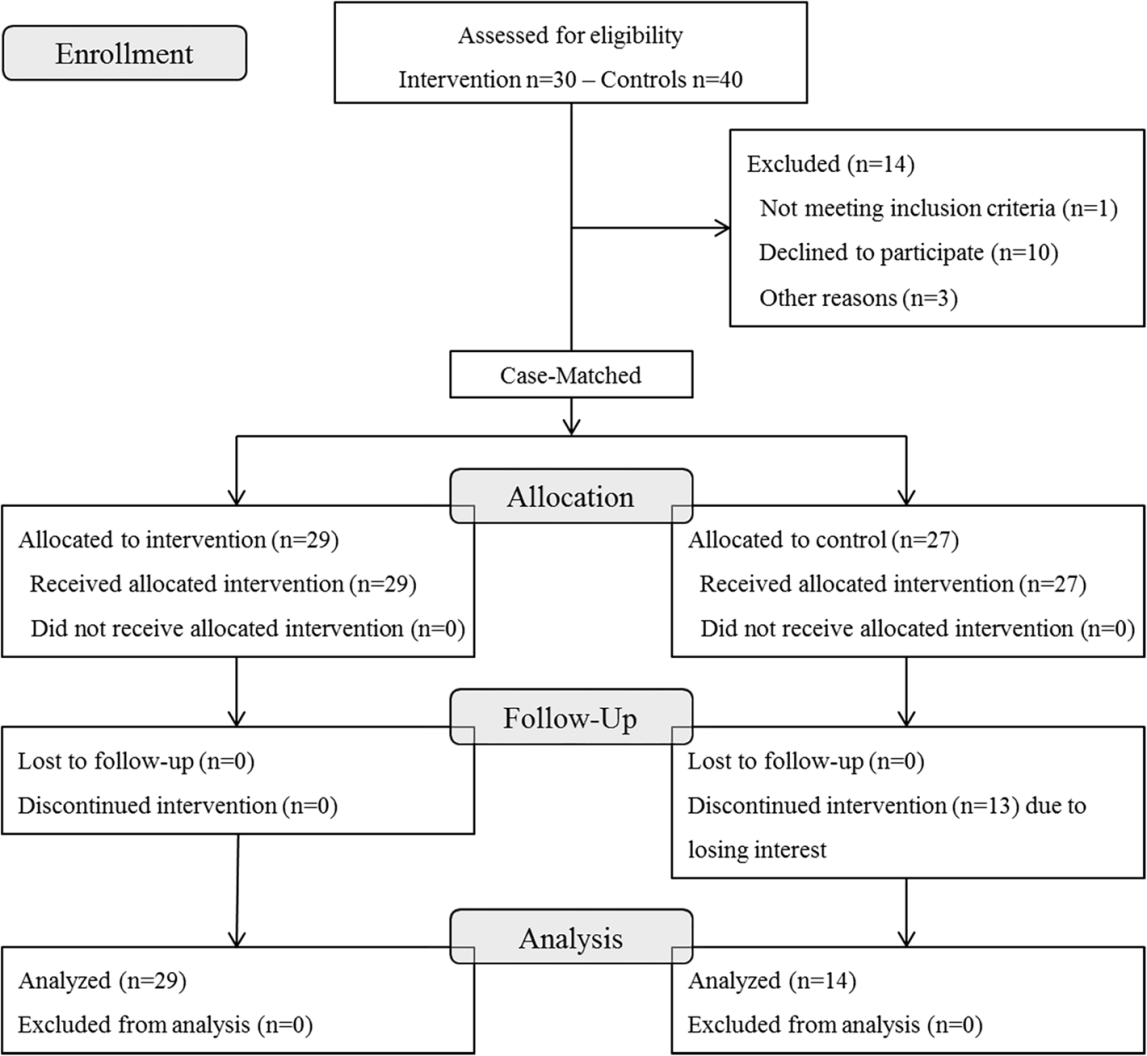
Flow diagram of study subject recruitment

Figure 1 presents a consort flow diagram with the details of enrollment, allocation, and analyses. In total, 70 patients with RA were first recruited into the study. Fourteen patients dropped out: one from the Tai Chi exercise group and 13 from the control group after the baseline assessment (Fig. 1). The 56 patients with RA were assigned to two groups by their willingness to participate, for which age and body mass index were matched; 29 patients in the Tai Chi exercise group and 27 patients in the control group that were given information about lifestyle modification, including smoking cessation and weight reduction. Patients in control group also received advice about appropriate regular exercises.

This study was approved by the ethics committee of Hanyang University Guri Hospital (2012-024). The procedures were fully explained to all subjects, and written informed consent was obtained.

### Exercise program

Patients participated in a Tai Chi exercise program “Twelve Movement Tai Chi for arthritis” [22]. This style applies small to large degrees of motion; knee flexion; straight and extended head and trunk; combined rotation of head, trunk, and extremities; and symmetrical diagonal arm and leg movements [23]. The program allows adjustment for movements to the functional level of the participant and within the comfort zone of either standing or sitting. The intervention was implemented as a group exercise once a week for 60 min over the course of 3 months at the hospital gymnasium.

### Assessments

#### Demographic and anthropometric data

Demographic data were collected by using a self-administered questionnaire. Eligible patients attended a rheumatology unit to undergo assessment by a trained nurse in the following standardized sequence: self-completed patient questionnaire, height, weight, and waist and hip circumference.

#### RA assessment

Clinical data were collected by means of interviews and clinical examination. The practitioners who performed clinical assessment were blinded to exercise group and study phase. Laboratory data to identify characteristics of RA were obtained. Anti-cyclic citrullinated peptide antibody (anti-CCP) was assayed by using the ImmuLisa CCP ELISA test (IMMCO Diagnostics Inc., Buffalo, NY, USA). A level of more than 25 units/ml of anti-CCP was considered positive. Contemporary inflammation was evaluated by the erythrocyte sedimentation rate (ESR) and C-reactive protein. The routine assessment of patient index data 3 (RAPID3)—remission (<3) and low (3.1–12) and high (>12) activity—and Disease Activity Score-28 (DAS-28) from visual analogue scale score of the patient’s global health, ESR, and number of swollen and tender joints—remission (<2.6) and low (2.6–5.1) or high (>5.1) activity—were calculated to assess disease activity. The Health Assessment Questionnaire (HAQ) was used to assess functional disability [24]. General clinical and RA medication information was also obtained.

#### Individual cardiovascular disease risk factors

Blood pressure was assessed following at least 5 min of rest, on either arm with the patient in a seated position. A venous blood sample was drawn after overnight fast to determine the following parameters: glucose, creatinine, total cholesterol, high-density lipoprotein cholesterol, triglyceride, and HbA1c. Smoking and comorbidity status were obtained from all patients via questionnaire.

#### Carotid intima-media thickness

Ultrasonographic evaluations for carotid intima-media thickness (cIMT) were achieved with an 11.3-MHz high-resolution linear probe and with simultaneous electrocardiographic recording. cIMT was measured at the T wave of the cardiac cycle on the common carotid wall at 1 cm from the bifurcation, calculating the distance between the intima-lumen interface and the media-adventitia interface. cIMT was measured bilaterally, and the average was determined for statistical analyses.

#### Flow-mediated dilatation

Brachial artery FMD was measured by using two-dimensional ultrasonography (iE33; Philips Medical Systems, Bothell, WA, USA) with a linear 11.3-MHz high-resolution probe. Measurements were taken with the patient supine for at least 10 min in a quiet room after an overnight fast; morning medications were not taken. The brachial artery was visualized in a longitudinal section, and baseline B-mode and Doppler images were obtained and diameter measurements were made. Subsequently, an adult blood pressure cuff was placed on the upper arm and inflated to 50 mm Hg above the systolic blood pressure for 5 min. Vasodilatory capacity was expressed as the percentage of change in the brachial artery diameter from baseline to 60 seconds post-cuff deflation. All images were digitized and recorded for study measurements, and techniques followed published guidelines [25]; these measures were interpreted by a single reader blinded to treatment and study phase. All patients abstained from smoking and drinking alcohol and coffee for at least 12 hours.

#### Brachial-ankle pulse wave velocity

After the subjects had rested in a supine position for more than 5 min, the measurement of baPWV was conducted by using a wave form analyzer (VP-2000; Colin Co Ltd., Komaki, Japan). Pulse waves were recorded automatically by sensors in the cuffs. The transmission times and distances between the cuffs on arms and legs were recorded, and the baPWV was produced as an output. The mean of the baPWVs in the left and right side was used for analysis.

### Statistical analysis

Normality of data distribution was evaluated with the Kolmogorov-Smirnov test. Accordingly, either a Student *t* test or a Mann-Whitney *U* test was used to compare continuous variables between groups (Tai Chi exercise versus control) at baseline. Chi-square tests were used to compare categorical variables between the two groups at baseline. The differences between values of the variables at baseline and those at the end of the 3-month Tai Chi exercise intervention were also compared by using either a Student *t* test or a Mann-Whitney *U* test. Analysis of covariance (ANCOVA) was employed to estimate the effect of Tai-Chi exercise on the changes of FMD and baPWV (dependent variables), independent of covariates which had also significantly changed over the period. To estimate the direct effects of Tai Chi exercise on outcome variables that changed significantly over time, we also performed mediation analysis by using a bootstrapping technique with bias-corrected confidence interval estimates. Mediation analysis using a bootstrapping technique is a statistical method that can effectively generate confidence interval estimates of the regression coefficients of independent variables that allow estimation of the direct and indirect effect of an independent variable on a dependent variable [26]. All statistical analyses were performed by using SPSS Statistics 21.0 (IBM SPSS 21.0; IBM Corporation, Chicago, IL, USA). Mediation analysis was performed with a set of 1,000 bootstrap resamples by using the PROCESS module incorporated in the SPSS software (model type 4, by Andrew F. Hayes, downloadable at http://www.afhayes.com).

## Results

Among the initial allocated 56 patients, 13 patients in the control group dropped out due to losing interest in the study. In total, 43 patients were further analyzed: Tai chi exercise group (n = 29) and control group (n = 14). Participants’ baseline characteristics are shown Table 1. The demographic, anthropometric characteristics between groups were similar. The mean ages ± standard deviations (SDs) (the mean disease duration, age ± SD) of the Tai Chi exercise group and control group were 64.0 ± 5.4 years (10.3 ± 9.4) and 60.9 ± 7.2 years (15.3 ± 7.8), respectively. There was a relatively high prevalence of medical history of metabolic disorders in each of these groups, and mean prevalence rates were 53.5 % (23/43) for hypertension, 7.0 % (3/43) for diabetes, and 46.5 % (20/43) for dyslipidemia. Anti-CCP positivity was 90.7 % in total patients with RA. According to their DAS-28 and RAPID3, in both groups, most patients with RA had low disease activity—mean DAS-28-ESR and RAPID3 scores of 3.8 and 9.4 (Tai Chi exercise group) and 3.5 and 9.0 (control group)—which were similar in the two groups. However, the number of tender joints (4.5 ± 5.5 versus 1.6 ± 1.5, *P* = 1.23 × 10^−2^) and functional disability score (HAQ) (0.63 ± 0.50 versus 0.35 ± 0.29, *P* = 2.43 × 10^−2^) were significantly higher in the Tai Chi exercise group compared with the control group, respectively. There were no significant differences between groups at baseline in any of the assessed atherosclerosis markers (cIMT, FMD, or baPWV).

**Table 1:** Baseline demographic, anthropometric, RA-related, and CVD characteristics, and atherosclerotic measurements for the total RA population as well as the exercise and control groups

	Total (n = 43)	Tai Chi group (n = 29)	Control group (n = 14)	*P*
Demographic, mean ± SD				
Age, years	63.6 ± 5.53	64.0 ± 5.4	62.7 ± 5.9	0.469
Anthropometric, mean ± SD				
Height, cm	153.8 ± 5.6	154.2 ± 5.7	153.0 ± 5.6	0.506
Weight, kg	54.0 ± 7.8	53.7 ± 7.6	54.6 ± 8.4	0.725
BMI, kg/m^2^	22.8 ± 2.9	22.5 ± 2.7	23.4 ± 3.2	0.388
Waist circumference, cm	79.3 ± 8.4	78.3 ± 8.5	81.4 ± 8.1	0.259
Waist-hip ratio	0.86 ± 0.53	0.85 ± 0.01	0.87 ± 0.05	0.142
Medical history, n (%)				
Hypertension	23 (53.5)	15 (51.7)	8 (57.1)	0.739
Diabetes mellitus	3 (7.0)	1 (3.4)	2 (14.3)	0.191
Dyslipidemia	20 (46.5)	12 (41.4)	8 (57.1)	0.331
Coronary artery disease	2 (4.6)	1 (3.4)	1 (7.1)	0.590
Cerebrovascular disease	4 (9.3)	2 (6.9)	2 (14.3)	0.434
Smoking	6 (13.9)	2 (6.9)	4 (28.6)	0.133
RA medication, n (%)				
Methotrexate	36 (83.7)	24 (82.8)	12 (85.7)	0.806
Hydroxychloroquine	17 (39.5)	11 (37.9)	6 (42.9)	0.757
Sulfasalazine	5 (11.6)	3 (10.3)	2 (14.3)	0.706
Leflunomide	11 (25.6)	7 (24.1)	4 (28.6)	0.755
Glucocorticoids	31 (72.1)	21 (72.4)	10 (71.4)	0.946
Dosage, median, mg/day	2.5	2.5	2.5	0.168
RA characteristics, mean ± SD				
Disease duration, years	12.0 ± 9.2	10.3 ± 9.4	15.4 ± 8.0	0.092
anti-CCP, n (%)	38 (88.4)	24 (82.8)	14 (100)	0.307
Swollen joint count, 0–69 joints	1.2 ± 2.4	1.5 ± 2.9	0.6 ± 0.9	0.237
Tender joint count, 0–69 joints	3.6 ± 4.7	4.5 ± 5.5	1.6 ± 1.5	0.012
DAS-28-ESR	3.7 ± 1.0	3.8 ± 1.1	3.5 ± 0.6	0.229
RAPID3	9.4 ± 4.5	9.4 ± 4.7	9.2 ± 4.0	0.895
HAQ	0.54 ± 0.46	0.63 ± 0.50	0.35 ± 0.29	0.024
ESR	31.7 ± 21.9	31.4 ± 22.7	32.2 ± 21.0	0.908
CRP	0.44 ± 0.44	0.48 ± 0.49	0.36 ± 0.34	0.384
CVD, mean ± SD				
Systolic BP, mm Hg	130.5 ± 11.6	129.2 ± 12.1	133.1 ± 10.5	0.313
Diastolic BP, mm Hg	77.6 ± 5.2	77.0 ± 4.8	78.7 ± 6.0	0.331
Heart rate	72.0 ± 6.1	72.5 ± 5.4	71.0 ± 7.5	0.473
Total cholesterol, mg/dl	184.6 ± 35.8	187.3 ± 36.1	179.0 ± 35.6	0.480
Triglycerides, mg/dl	112.2 ± 47.4	108.7 ± 36.6	122.14 ± 54.3	0.343
HDL, mg/dl	61.6 ± 12.0	63.9 ± 12.1	56.8 ± 10.5	0.070
LDL, mg/dl	97.9 ± 31.1	96.6 ± 31.4	100.7 ± 31.7	0.689
Fasting glucose, mg/dl	91.5 ± 11.1	89.8 ± 9.3	95.1 ± 13.9	0.141
Creatinine, mg/dl	0.74 ± 0.12	0.72 ± 0.12	0.76 ± 0.11	0.300
Atherosclerosis, mean ± SD				
cIMT, mm	0.68 ± 0.13	0.69 ± 0.13	0.66 ± 0.13	0.596
FMD, %	6.00 ± 2.06	5.85 ± 2.05	6.31 ± 2.12	0.494
baPWV, cm/s	1708.8 ± 303.3	1693.7 ± 348.3	1740.3 ± 185.3	0.643

Values are presented as mean ± standard deviation (*SD*). *anti-CCP* anti-cyclic citrullinated peptide antibody, *BMI* body-mass index, *DAS-28* disease activity score-28, *ESR* erythrocyte sedimentation rate, *CRP* C-reactive protein, *RAPID3* routine assessment of patient index data 3, *HAQ* Health Assessment Questionnaire, *BP* blood pressure, *HDL* high-density lipoprotein, *LDL* low-density lipoprotein, *cIMT* carotid intima-media thickness, *FMD* flow-mediated dilatation, *baPWV* brachial-ankle pulse wave velocity

### Effects of Tai Chi exercise on disease activity of RA and CVD characteristics

Results of change in body composition, RA-related characteristics, and cardiovascular risk factors after the 3-month follow-up are summarized in Table 2. All changes (Δ: initial – at 3 months) of RA characteristics, including disease activity (mean ΔDAS-28-ESR and ΔRAPID3) and functional disability (mean ΔHAQ), related to RA over time were not significantly different between the two groups. Blood pressure and heart rate were not significantly changed in the two groups. Interestingly, total cholesterol after the 3-month follow-up significantly decreased in the Tai Chi exercise group compared with the control group: mean Δ total cholesterol −7.8 (Tai Chi group) and 2.9 (control group) mg/dl, *P* = 2.72 × 10^−2^.

**Table 2:** Changes in body composition, rheumatoid arthritis-related characteristics and cardiovascular risk factors at 3-month follow-up

	Tai Chi group (n = 29)	Control group (n = 14)	*P*
Anthropometric, mean ± SD			
ΔBMI, kg/m^2^	0.0 ± 0.63	−0.1 ± 1.52	0.530
ΔWaist circumference, cm	−0.2 ± 3.7	0.1 ± 4.0	0.537
RA characteristics, mean ± SD			
ΔSwollen joint count, 0 ~ 69 joints	−0.6 ± 3.3	0.0 ± 1.7	0.834
ΔTender joint count, 0 ~ 69 joints	−2.5 ± 4.5	0.1 ± 3.0	0.107
ΔDAS-28-ESR	−0.4 ± 1.1	−0.0 ± 1.1	0.247
ΔRAPID3	−2.2 ± 4.7	−1.0 ± 3.9	0.404
ΔHAQ	−0.13 ± 0.29	0.00 ± 0.20	0.274
ΔESR	2.0 ± 18.0	−1.1 ± 12.4	0.569
ΔCRP	0.2 ± 0.8	0.1 ± 0.6	0.399
CVD, mean ± SD			
ΔSystolic BP, mm Hg	−0.9 ± 10.5	1.8 ± 14.4	0.496
ΔDiastolic BP, mm Hg	0.2 ± 6.8	−0.1 ± 6.3	0.910
ΔHeart rate	1.3 ± 4.9	3.4 ± 4.4	0.238
ΔTotal cholesterol, mg/dl	−7.8 ± 15.5	2.9 ± 12.2	2.72 × 10^−2^
ΔTriglycerides, mg/dl	−2.48 ± 32.99	6.58 ± 36.18	0.078
ΔHDL, mg/dl	6.91 ± 12.82	0.53 ± 8.73	0.053
ΔLDL, mg/dl	−9.2 ± 13.2	−4.8 ± 12.6	0.337
ΔFasting glucose, mg/dl	2.3 ± 13.6	−5.6 ± 12.7	0.227
Atherosclerotic measurements, mean ± SD			
ΔcIMT, mm	−0.02 ± 0.08	−0.02 ± 0.08	0.746
ΔFMD, %	1.90 ± 2.00	−0.54 ± 2.49	1.76 × 10^−3^
ΔbaPWV, cm/s	−93.6 ± 152.2	52.6 ± 224.1	1.57 × 10^−2^

Values are presented as mean ± standard deviation (*SD*). *BMI* body-mass index, *DAS-28* disease activity score-28, *ESR* erythrocyte sedimentation rate, *CRP* C-reactive protein, *RAPID3* routine assessment of patient index data 3, *HAQ* Health Assessment Questionnaire, *BP* blood pressure, *HDL* high-density lipoprotein, *LDL* low-density lipoprotein, *cIMT* carotid intima-media thickness, *FMD* flow-mediated dilatation, *baPWV* brachial-ankle pulse wave velocity

### Effects of Tai Chi on endothelial function and arterial stiffness

**Fig. 2:**
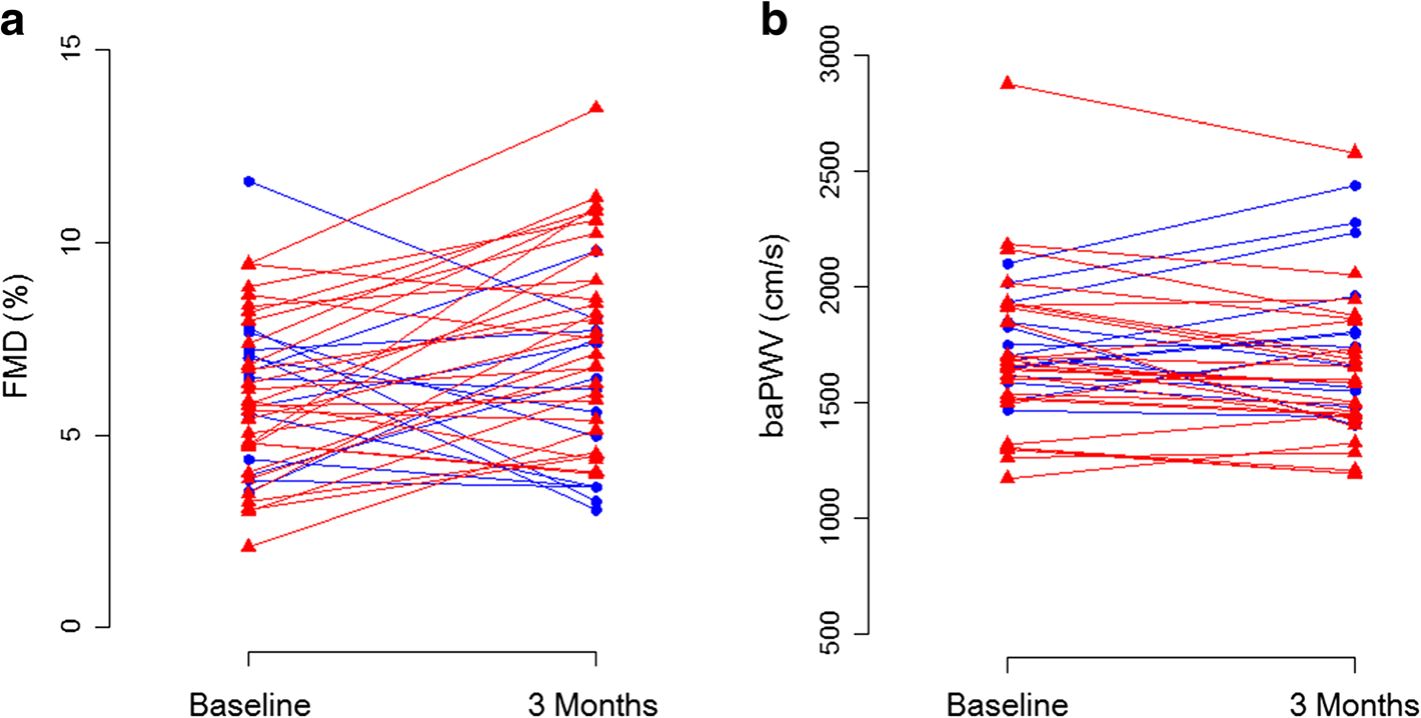
Change in flow-mediated dilatation (FMD) (**a**) and brachial-ankle pulse wave velocity (baPWV) (**b**) from baseline to 3 months for Tai Chi exercise group (*red*) versus control group (*blue*)

Changes in FMD over time were significantly different between the two groups (*P* = 1.76 × 10 − ^3^), with a significant increment in the Tai Chi exercise group—initial versus 3 months (5.85 ± 2.05 versus 7.75 ± 2.53 %), mean ΔFMD 1.90 %—but not in the control group: initial versus 3 months (6.31 ± 2.12 versus 5.78 ± 2.13 %), mean ΔFMD −0.50 % (Table 2 and Fig. 2a). Changes in baPWV over time were significantly different between the two groups (*P* = 1.57 × 10^−2^), with a significant reduction in the Tai Chi exercise group (1693.7 ± 348.3 versus 1600.1 ± 291.0 cm/s, mean Δ baPWV −93.6 cm/s) but not in the control group (1740.3 ± 185.3 versus 1792.8 ± 326.1 cm/s, mean Δ baPWV 52.6 cm/s) (Table 2 and Fig. 2b). In addition, these changes in FMD and baPWV were significantly negatively correlated (r = −0.33, *P* = 0.031) (Additional file 1). However, the change in cIMT was not significantly different between the Tai Chi exercise and control groups (*P* = 0.746).

Tai Chi exercise was associated with a significant improvement in FMD and baPWV as well as total cholesterol. We examined the significance of Tai Chi exercise regarding ΔFMD and ΔPWV as dependent variables by using an ANCOVA model that included Δtotal cholesterol. After adjustment for Δtotal cholesterol, Tai Chi exercise was associated with ΔFMD (*P* = 4.32 × 10^−3^) and ΔbaPWV (*P* = 2.22 × 10^−2^) (Table 3). Moreover, since the improvement in total cholesterol level could have positively impacted on the change of FMD and baPWV, we performed mediation analysis by using the bootstrapping method with bias-corrected confidence interval estimates to evaluate whether the change in total cholesterol level was associated with ΔFMD and ΔbaPWV. Tai Chi exercise modestly correlated with Δtotal cholesterol (r = 0.27, *P* = 7.76 × 10^−2^), but mediation analysis showed that the indirect effects of Tai Chi exercise on ΔFMD and ΔbaPWV through Δtotal cholesterol were not significant (β coefficient, 95 % CI = 0.13, −0.19 to 0.82 for ΔFMD and −1.0, −53.1 to 25.6 for ΔbaPWV), whereas the direct effects of Tai Chi exercise were significant (β coefficient, 95 % CI = 2.31, 0.77 to 3.86, *P* = 4.32 × 10^−3^ for ΔFMD and −145.1, −268.4 to −21.8, *P* = 2.22 × 10^−2^ for ΔbaPWV) (Additional file 2).

**Table 3:** Analysis of covariance analysis for the determinants of ∆FMD and ∆baPWV

	Independent variables	β coefficient	S.E.	*P* value
∆FMD	Intercept	−0.525	0.604	
	Tai Chi	2.312	0.764	4.32 × 10^−3^
	∆Cholesterol	−0.015	0.024	0.532
∆baPWV	Intercept	52.456	48.456	
	Tai Chi	−145.121	61.018	2.22 × 10^−2^
	∆Cholesterol	0.117	1.9	0.951

*FMD* flow-mediated dilatation, *baPWV* brachial-ankle pulse wave velocity, *S.E.* standard error

## Discussion

Our findings show that Tai Chi exercise in elderly women with RA significantly improves endothelial dysfunction and arterial stiffness, which are known atherosclerosis precursors, useful indexes for early detection of CVD, and predictors for increased cardiovascular mortality [27–30]. To the best of our knowledge, this study is the first evidence of a possible reduction of cardiovascular risk through Tai Chi exercise by improving endothelial dysfunction and arterial stiffness in patients with RA.

The increased risk of CVD in patients with RA has recently become the focus of intense investigations. In RA, disease-related inflammation and traditional risk factors, such as hypertension, diabetes, dyslipidemia, and smoking, are widely assumed to contribute to the elevated CVD risk [7–9]. Moreover, physical inactivity is likely a notable risk factor in RA. Therefore, exercise can be an important behavioral strategy for CVD prevention in patients with RA as well as in the general population [31]. Moreover, exercise is a cost-effective intervention that may significantly improve cardiorespiratory fitness, CVD risk factors, and 10-year CVD event probability in RA [31–33].

Recently, there has been increasing interest in the promotion of aerobic exercise or increasing physical activity for patients with RA, although many clinicians still discourage such activity because of concerns about exacerbating joint damage [34]. Because many patients with RA have below-average physical capacity and lead a sedentary lifestyle, low-intensity exercise tailored to individual needs is recommended for patients with RA [35]. During the past several years, multiple trials have been completed to examine the effect of exercise on RA, but most focus on pain, disease activity, functional ability, quality of life, structural damage, and aerobic capacity [2, 32, 34, 36–39]. Although one study found that an individualized exercise training program, which consisted of a 6-month tailored aerobic and resistance exercise intervention, improved endothelial function in patients with RA [18], there are limited data available regarding the optimal dose and types of exercise as well as the cardioprotective effects of exercise in RA.

Tai Chi is a Chinese martial art that combines meditation with slow, gentle, graceful movements as well as deep breathing and relaxation [40]. Intensity in Tai Chi is low and equivalent to walking 6 km/h and provides a moderate increase in heart rate [41]. Significant improvement in cardiopulmonary function has been found in Tai Chi practitioners when compared with sedentary control subjects of middle age and older [42–44]. Tai Chi training can also improve cardiopulmonary function in patients with CVD, such as chronic heart failure [45, 46] and myocardial infarction [47]. Recent studies have reported that Tai Chi has been found to improve arterial compliance in elderly subjects [48, 49]. Recently, Tai Chi has been applied with substantial benefits in patients with RA. Tai Chi leads to reduced disability and fatigue [20, 50] and is considered safe in patients with RA, especially long-standing and dramatically physically inactive individuals [51]. A Cochrane review on Tai Chi exercise concluded that there were positive effects on a selected range of motion outcomes as well as increased level of participation and enjoyment of exercise for patients with RA [52]. Moreover, recent systematic reviews have shown that Tai Chi can reduce blood pressure and increase cardiovascular exercise capacity in patients with CVD and cardiovascular risk factors [53]. Despite encouraging evidence suggesting that Tai Chi has multiple benefits for patients with RA, few studies have reported relationships between Tai Chi exercise and any other cardiovascular risk factors or surrogate markers of atherosclerosis. In the present study, we demonstrated that Tai Chi exercise significantly improved endothelial function, arterial stiffness, and lipid profile, suggesting that Tai Chi exercise can be a useful behavioral strategy for CVD prevention as well as for promotion of aerobic exercise and physical activity in patients with RA.

Aerobic exercise has been shown to improve endothelial dysfunction [54, 55] and arterial stiffness [16, 56], but the mechanism by which Tai Chi exercise improves these functions remains unclear. One suggestion is that the meditative components of Tai Chi have the potential to reduce stress levels, which can mediate a range of effects by attenuating the sympathoadrenal axis. Reductions in catecholamine levels can improve the lipid profile, the hemodynamic profile, including blood pressure, and the coagulation profile [57, 58]. Similarly, stress can activate the hypothalamic-pituitary-adrenal axis, increasing hypothalamic release of multiple corticotrophin secretagogues, corticotrophin-releasing hormone, and arginine vasopressin. Cortisol hypersecretion has been associated with hypertension and the development of the constellation of cardiovascular risk factors, including diabetes, hypertension, and dyslipidemia, termed the metabolic syndrome, and associated cardiovascular comorbidities [59]. Another suggestion is that Tai Chi decreased sympathetic nervous system activity in older adults [60] and could improve baroreflex sensitivity and heart rate variability in patients with coronary heart disease [61], which are closely associated with endothelial dysfunction and arterial stiffness and are predictors of mortality in patients with coronary heart disease.

Interestingly, participants in the Tai Chi exercise group showed an improvement in their lipid profile, possibly indicating a favorable effect of Tai Chi exercise on the lipid metabolism. Our findings are in line with previous studies that examined changes in lipid profile as a result of Tai Chi exercise [58, 62]. As all participants were informed of their higher risk profile and were given general information about lifestyle modification and exercise, dietary change or lifestyle modification during the exercise training period would be expected to have favorable effects on total cholesterol, but the mechanisms by which Tai Chi exercise may improve lipid profiles remain uncertain. The possibility that the change in body fat ratio and insulin resistance might have an influence on lipid profile should be considered. Favorable changes in lipid profile after Tai Chi exercise likely play a role in improving endothelial dysfunction and arterial stiffness. To examine the mediation effect of Δtotal cholesterol on ΔFMD and ΔPWV after Tai Chi exercise, we constructed a bootstrapping mediation analysis. As expected, these data further support the notion that Tai Chi exercise may lower cardiovascular risk in RA patients via a beneficial effect on the arterial wall, independently of the improvement in lipid profile.

There are a few limitations in our study. The sample size was small, and there were a considerable number of control-group participants who were lost to follow-up. In this prospective observational study, we recruited only elderly women with RA and so the results may not be applicable to men or all patients with RA. Only short-term follow-up assessment was performed and so no long-term effects could be established. The present data indicate that, for the cohort studied, the positive profiles of change in arterial stiffness and endothelial function were not significantly related to improvements in RA disease activity or blood pressure. We are unable to specify whether these improvements in vascular function are the direct consequence of the Tai Chi exercise since no assessments about physical activity, which was also known to reduce cardiovascular risk factors and associated mortality in patients with sedentary lifestyle, were included in the study. We are also unable to document whether perceived benefits from the Tai Chi exercise related to the improved cardiovascular outcome. It is recommended that such measures be included in future studies to elucidate the mechanism for improvements resulting from Tai Chi exercise. Moreover, further large-scale, long-term randomized clinical trials should be needed to determine whether and to what extent improvement of endothelial dysfunction and arterial stiffness by Tai Chi exercise affects disease activity as well as clinical cardiovascular events in patients with RA. Despite these limitations, positive outcomes reported in the present study provide the rationale for the wider adoption of Tai Chi exercise as a health-promoting activity in patients with RA and to help overcome individual barriers to exercise, especially among long-standing and elderly patients with RA.

## Conclusions

This study demonstrated that Tai Chi exercise significantly contributed to improvement in endothelial function and arterial stiffness, independently of the traditional CVD risk factors, including lipid profile. Tai Chi exercise may be a useful treatment strategy for RA as a complementary and alternative medical approach to preventing CVD.

## Ethics approval

This study was approved by the institutional review board of Hanyang University.

## Patient consent

Consents were obtained from all patients who participated in this study.

## Additional files

Additional file 1:
**Correlation plot of change in flow-mediated dilatation (FMD) versus change in brachial-ankle pulse wave velocity (baPWV) after 3 months of Tai Chi exercise.** (JPG 337 kb) Additional file 2:
**Mediation analysis to evaluate whether the change in total cholesterol level was associated with the change in flow-mediated dilatation (FMD) and brachial-ankle pulse wave velocity (baPWV) after Tai Chi exercise.** (JPG 481 kb)

## Abbreviations

ANCOVA: Analysis of covariance
anti-CCP: Anti-cyclic citrullinated peptide antibody
baPWV: Brachial-ankle pulse wave velocity
cIMT: Carotid intima-media thickness
CVD: Cardiovascular disease
DAS-28: Disease activity score-28
DMARD: Disease-modifying anti-rheumatic drug
ESR: Erythrocyte sedimentation rate
FMD: Flow-mediated dilatation
HAQ: Health Assessment Questionnaire
RA: Rheumatoid arthritis
RAPID3: Routine assessment of patient index data 3
SD: Standard deviation
