# Supplementary figures and images for: The beneficial effects of Tai Chi exercise on endothelial function and arterial stiffness in elderly women with rheumatoid arthritis
Source: Arthritis Res Ther. 2015 Dec 24;17:380. doi: 10.1186/s13075-015-0893-x (PMC4718020; doi:10.1186/s13075-015-0893-x)

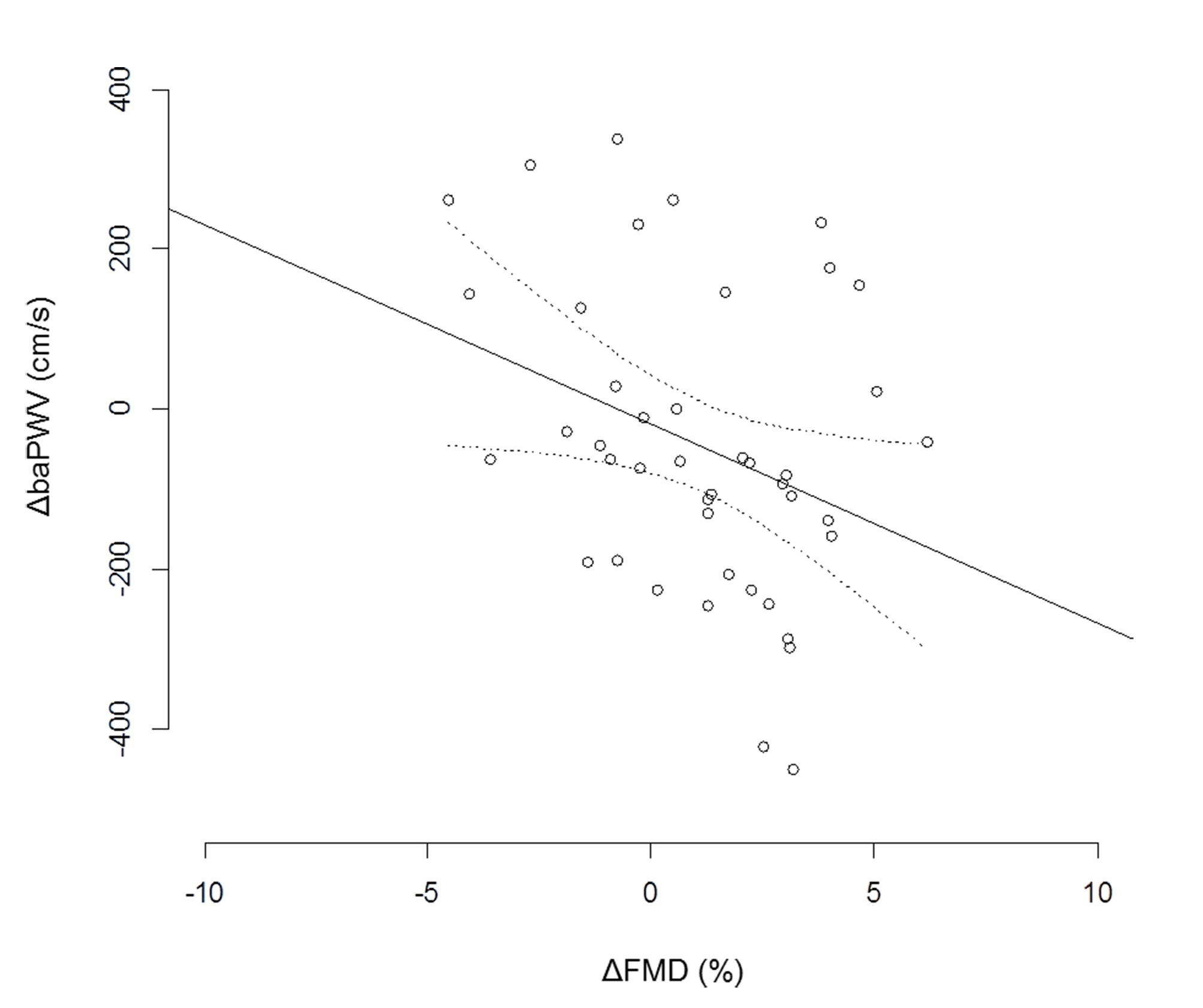

Supplement: Additional file 1: — Correlation plot of change in flow-mediated dilatation (FMD) versus change in brachial-ankle pulse wave velocity (baPWV) after 3 months of Tai Chi exercise. (JPG 337 kb) [file 13075_2015_893_MOESM1_ESM.jpg]

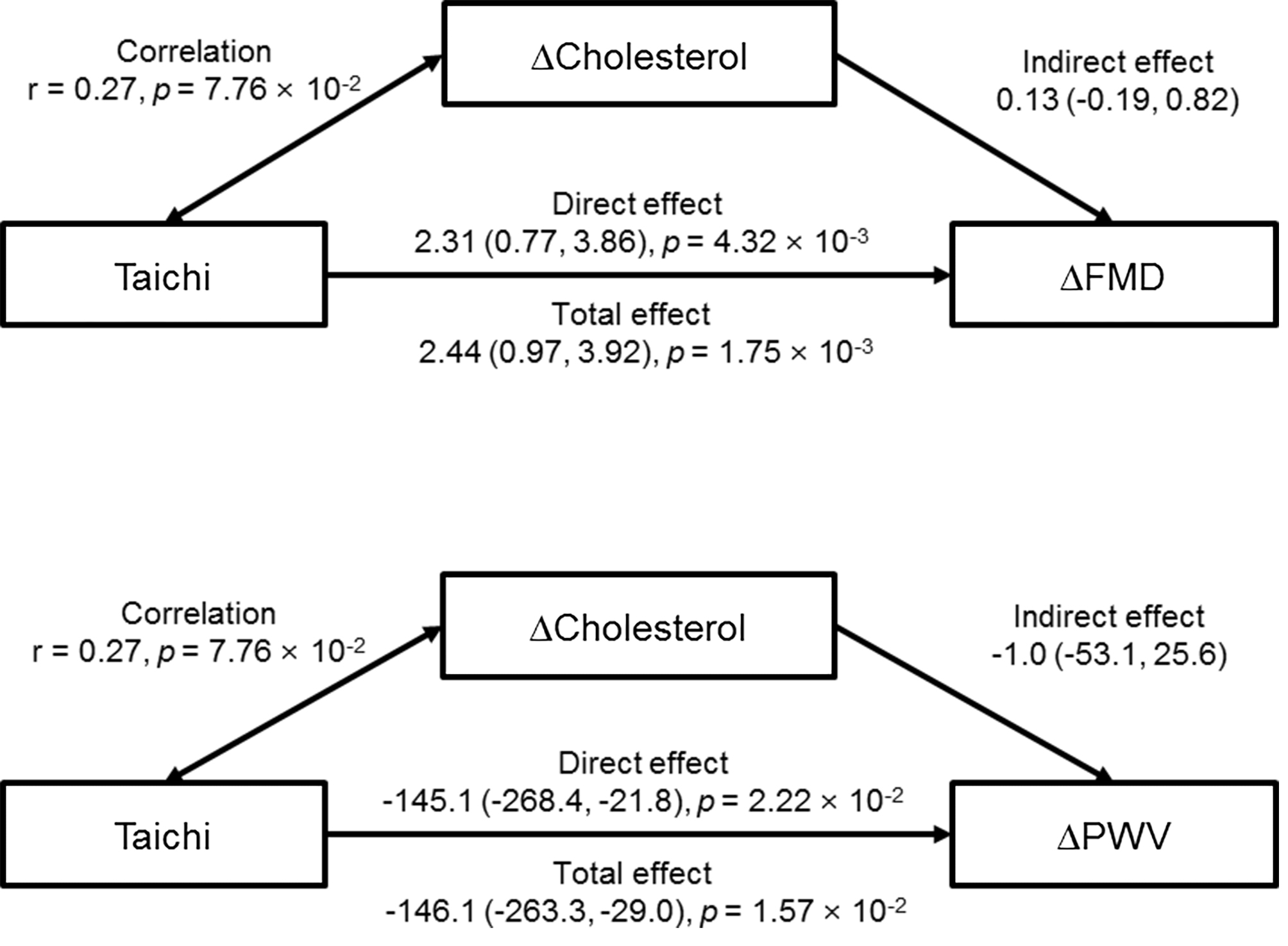

Supplement: Additional file 2: — Mediation analysis to evaluate whether the change in total cholesterol level was associated with the change in flow-mediated dilatation (FMD) and brachial-ankle pulse wave velocity (baPWV) after Tai Chi exercise. (JPG 481 kb) [file 13075_2015_893_MOESM2_ESM.jpg]
